# Effects of a phytogenic, alone and associated with potassium diformate, on tilapia growth, immunity, gut microbiome and resistance against francisellosis

**DOI:** 10.1038/s41598-019-42480-8

**Published:** 2019-04-15

**Authors:** S. A. Suphoronski, R. T. Chideroli, C. T. Facimoto, R. M. Mainardi, F. P. Souza, N. M. Lopera-Barrero, G. F. A. Jesus, M. L. Martins, G. W. Di Santis, A. de Oliveira, G. S. Gonçalves, R. Dari, S. Frouel, U. P. Pereira

**Affiliations:** 1 Laboratory of Fish Bacteriology (LABBEP) - Department of Preventing Veterinary Medicine, State University of Londrina, Londrina, PR Brazil; 2 Department of Animal Science, State University of Londrina, Londrina, PR Brazil; 3 Nucleus of studies in Aquaculture Pathology, Federal University of Santa Catarina, Florianópolis, SC Brazil; 4 Laboratory of Microbial Biotechnology (LABIM) and Laboratory of Electron Microscopy and Microanalysis, State University of Londrina, Londrina, PR Brazil; 5 Fishing Institute (APTA-SP), São José do Rio Preto, SP Brazil; 6 MiXscience, Bruz, Brittany France

## Abstract

This work evaluated the effects of dietary supplementation of A-Live (phytogenic) either individually or in combination with Aquaform (potassium diformate, acidifier) on juvenile Nile tilapia (*Oreochromis niloticus*) growth performance, innate immune parameters, gut microbiome, and resistance against *Francisella noatunensis* subsp. *orientalis* challenge. Each experimental group contained 140 fishes (34.3 ± 0.33) in two 150L tanks. The experimental design consisted of five groups: a negative control; treated groups (G1, G2, G3) supplemented with different concentrations of A-Live and Aquaform in the feed; and a positive control (PC) for pathogen infection. Groups G1, G2, G3, and PC were challenged with *Francisella* spp. after 15 days. After infection, the mortality was significantly lower in groups G1, G2, and G3 (p < 0.01). Furthermore, these groups showed significant increase (p < 0.05) in daily weight gain, feed conversion rate, and specific growth rate. The PC group presented increase (p < 0.05) in the leukocytes and neutrophils number. Innate immunity parameters showed no difference between treatments after infection. Microbiome analysis revealed an increased number of bacteria belonging to the Vibrionaceae family after pathogen infection suggesting a secondary pathogen function of these bacteria. These results validate the beneficial effects of these products in tilapia farming.

## Introduction

World aquaculture is the sector of animal production that has been showing the highest rate of growth in recent decades^1–3^. Despite the high potential to increase production of aquatic organisms, there are still many barriers to the development of this sector in many countries^4–6^. This rapid growth has led producers to use intensive and super-intensive production systems that have positively increased productivity but have also increased the disease susceptibility of fish^7^. Some bottlenecks limit production in this sector, such as the availability of equipment and adequate rations, intensive production systems, environmental licenses, imports, low disclosure^8^ and outbreaks of infectious diseases, which are highlighted as one of the main problems of aquaculture, especially in fish farming^9,10^. Protozoa, viruses, fungi, and bacteria are the main etiological agents associated with mortality in tilapia farms. The bacterial disease outbreaks may decimate up to 90% of the affected batches^11–13^.

The main bacterial pathogens associated with Nile tilapia disease are *Streptococcus agalactiae*, *S*. *iniae*, *S*. *dysgalactiae*, *Flavobacterium columnare and Francisella noatunensis* subsp. *orientalis*^13–16^. In recent years, outbreaks caused by *Francisella* spp. have gained notoriety as the major pathogen of tropical fish cultivated worldwide^17,18^. Francisellosis is an emerging disease that has spread to several countries in the last two decades^17,18^ due to the absence of commercially available effective vaccines and difficulties in antibiotic treatment because the disease is caused by a facultative intracellular pathogen^19,20^.

Currently, the main therapeutic measure for francisellosis outbreaks is antibiotic therapy. However, its indiscriminate use may lead to the development of antibiotic resistance and poses potential risk to the environment, public health and food safety. In many cases, antibiotics (administered on feed) may have a limited efficacy when fish present hyporexia/anorexia, which is one of the first clinical signs of disease^19^ or due to the late onset of treatment after the loss of affected fish batches^21,22^. Therefore, the search for less harmful and more environmentally friendly treatments is of major importance.

Due to the intrinsic properties of dietary supplements such as phytogenic compounds combined with or without organic acids appear to be one of the most promising alternatives to prevent microbial infections^23–25^. Indeed, phytogenic compounds are defined as plant-derived natural bioactive compounds which show positive effects on animal growth and health, and they often encompass essential oils (EOs), botanicals and herbal extracts^24^. Recent studies have reported that phytogenic compounds are capable of promoting improved performance, stimulating the innate immune response, and decreasing mortality against bacterial challenges in fish. Common carps (*Cyprinus carpio*) supplemented with EOs of *Litsea cubeba* presented better growth performance and when challenged with *Aeromonas hydrophila* they had a higher relative survival than the control group^26^. Phytogenic compounds derived from *Ocimum gratissimum* and *O*. *americanum* have been shown to be more efficient in promoting increased activity of the complement system in catfish (*Rhamdia quelen*)^27^. Nevertheless, studies evaluating the effect of phytogenic compounds on growth, immunity, and resistance to diseases in Nile tilapia are rare.

Furthermore, organic acids (and their salts), such as formic, citric, acetic, propionic, and butyric acid, among others, are already used as acidifiers with success in the animal feed industry, including aquaculture^28–30^. The main function of these organic acids is the reduction of the pH of the digestive tract, kill pathogenic bacteria, reduce the digestion tract emptying time, increase pepsin activity and nitrogen retention, and improve the digestibility, absorption and transport of minerals^25^. However, the efficacy of these food supplements in the prevention and/or control of different bacterial diseases in tilapia (especially francisellosis) and their effect on the intestinal bacterial population has been poorly studied.

The combination of a phytogenic compound with antimicrobial activity and an organic acid seems to be an efficient and reliable alternative to antibiotic administration. Thus, the aim of this study was to measure the *in vitro* activity of phytogenic compound A-Live (MiXscience, France) individually or in combination with an organic acid Aquaform (Salus, Santo Antônio da Posse, SP, Brazil) against *Francisella noatunensis* subsp. *orientalis* and also evaluate the *in vivo* potential of the products treatment on growth performance, innate immunity, gut microbiome and francisellosis resistance in juvenile Nile tilapia (*Oreochromis niloticus*).

## Results

### *In vitro* antibacterial activity of products against *Francisella noatunensis* subsp. orientalis

Aquaform had inhibitory effects against *Francisella* spp. at concentrations ranging from 0.5 to 10%, showing halos of 13 to 20 mm. For A-live, inhibition values extended from 0 to 30 mm. The products, when used isolated, did not show inhibitory effects against the bacteria at a concentration of 0.1%. However, when used in combination, the products showed an inhibitory halo of 15 or 18 mm at a low concentration (0.1%) in 2 consecutive replicates.

**Figure 1:**
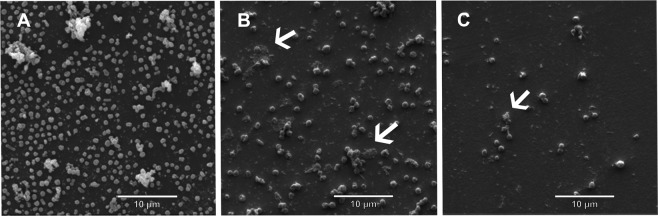
Visualization of number of cells of *Francisella* spp. F1 strain by electron microscopy after 30 min of exposure with both products applied either at 0.1% or 1%, (**A**) (*Francisella* spp. without the products); (**B**) (*Francisella* spp. with A-Live 0.1% and Aquaform 0.1%); (**C**) (*Francisella* spp. with A-Live 1% and Aquaform 1%). Cellular damages are shown using white arrows (**B**,**C**).

The results of electron microscopy showed a decline in the cell number of *Francisella* spp. after 30 min in the presence of either Aquaform or A-Live individually. However, a smaller number of bacterial cells were visualized in the presence of A-Live than in Aquaform, suggesting that A-Live was capable of causing rapid damage to *Francisella* spp. (Supplementary Fig. 1). Additionally, the combination of Aquaform and A-Live at a concentration of 0.1% (of each product) showed a reduction in the number of *Francisella* spp. cells and a large amount of destroyed cell fragments. The combination of products at 1% revealed a drastic reduction in cell number when compared to the cell number reduction in other concentrations of mixture of products (Fig. 1) or when treated individually (Supplementary Fig. 1).

### Growth performance and disease challenges

Prior to pathogen challenge, groups G2 and G3 showed a superior feed conversion ratio (FCR) and specific growth rate (SGR) than that in two groups that did not receive the products in the feed (NC and PC) and G1 group. The G2 group presented better daily weight gain and SGR than that in other groups. Group G1 showed similar results when compared with the PC and NC groups. Even after disease challenge, the G2 and G3 groups presented a better daily weight gain, FCR, and SGR than that of the PC and G1 groups but did not differ from that of the NC group (Table 1).

**Table 1:** Growth performance of fish from different treatments before and after disease challenge with *F*. *noatunensis* subsp. *orientalis* strain F1.

Analyzed period	Groups	Initial weight (g)	Final weight (g)	Daily weight gain (g)	Food consumption/fish/day (g)	Feed conversion rate (FCR)	Specific growth rate (SGR)
7 days of acclimation and 15 days of experimental treatment	NC	35	50.6	0.74^b^	1.63	2.19^b^	1.75^b^
	PC	36	51.1	0.72^b^	1.66	2.31^b^	1.66^b^
	G1	35	49.8	0.70^b^	1.56	2.21^b^	1.67^b^
	G2	35	56.9	1.04ª	1.93	1.85ª	2.31ª
	G3	33	52.7	0.94^ab^	1.71	1.82^a^	2.22^ab^
14 days post *Francisella* spp. Challenge	NC	50.6	60.7	0.72ª	1.01	1.40ª	1.29ª
	PC	51.1	57.4	0.45^b^	0.9	2.00^b^	0.83^b^
	G1	49.8	56	0.44^b^	0.77	1.74^b^	0.83^b^
	G2	56.9	66.3	0.67ª	0.9	1.34ª	1.09ª
	G3	52.7	61.25	0.61^a^	0.8	1.31ª	1.07ª

^*^Different letters (a and b) indicate significant difference between the treatments (p < 0.05).

NC, negative control; PC, positive control; G1, 0.2% A-Live treatment; G2, 0.2% A-Live and 0.2% Aquaform treatment; G3, 0.5% A-Live and 0.2% Aquaform.

**Figure 2:**
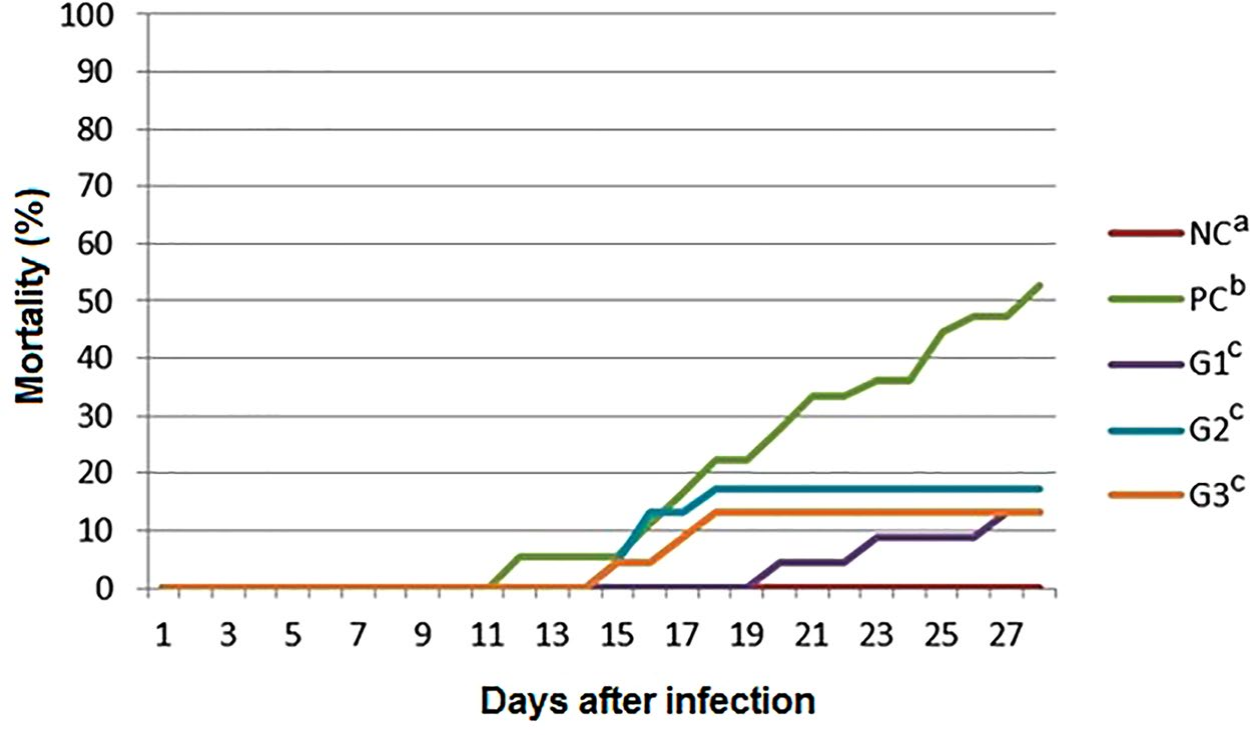
Cumulative mortality observed in the different groups after disease challenge by immersion with *Francisella noatunensis* subsp. *orientalis*. NC negative control: No challenge with bacteria and no A-Live or Aquaform (2 tanks); PC positive control: Challenge with *Francisella* spp. and no A-Live or Aquaform; G1- fish that received A-Live at 0.2% in the feed for 15 days prior to experimental infection with *Francisella* spp. (2 tanks); G2- fish that received the product A-Live at 0.2% and Aquaform at 0.2% in the feed for 15 days prior to experimental infection with *Francisella* spp.; G3- fish that received the product A-Live at 0.5% and Aquaform at 0.2% in the feed for 15 days prior to experimental infection with *Francisella* spp. (2 tanks).

The cumulative mortality of fishes in the group PC, G1, G2 and G3, which were pathogen challenged through feed, was 80, 65, 70, and 77%, respectively (Supplementary Fig. 2). Daily feed consumption was higher in groups G2 and G3 than in group G1 during the challenge period. Only G1 group showed a significant difference in cumulative mortality when compared to PC group (p < 0.05). In the pathogen challenge by immersion, cumulative mortality observed in PC, G1, G2 and G3 groups was 52, 13, 17 and 13%, respectively (Fig. 2). There was a significant difference in the mortality of G1, G2 and G3 groups compared with the mortality of the PC group (p < 0.0001).

Not all the surviving fish in the treated groups showed francisellosis lesions on the last day of the immersion experiment. In the PC and G1 groups, all surviving fish had granulomatous lesions in the cranial kidney and spleen. However, in the G2 and G3 groups, 15% and 35% of animals, respectively, did not exhibit francisellosis lesions in these organs. Additionally, these samples were negative in the CHAH culture. No lesions were observed in the kidney and spleen of the NC group. No mortality was observed in the oral or immersion negative control groups, indicating that the control was reliable.

### Intestine histology and morphology

**Figure 3:**
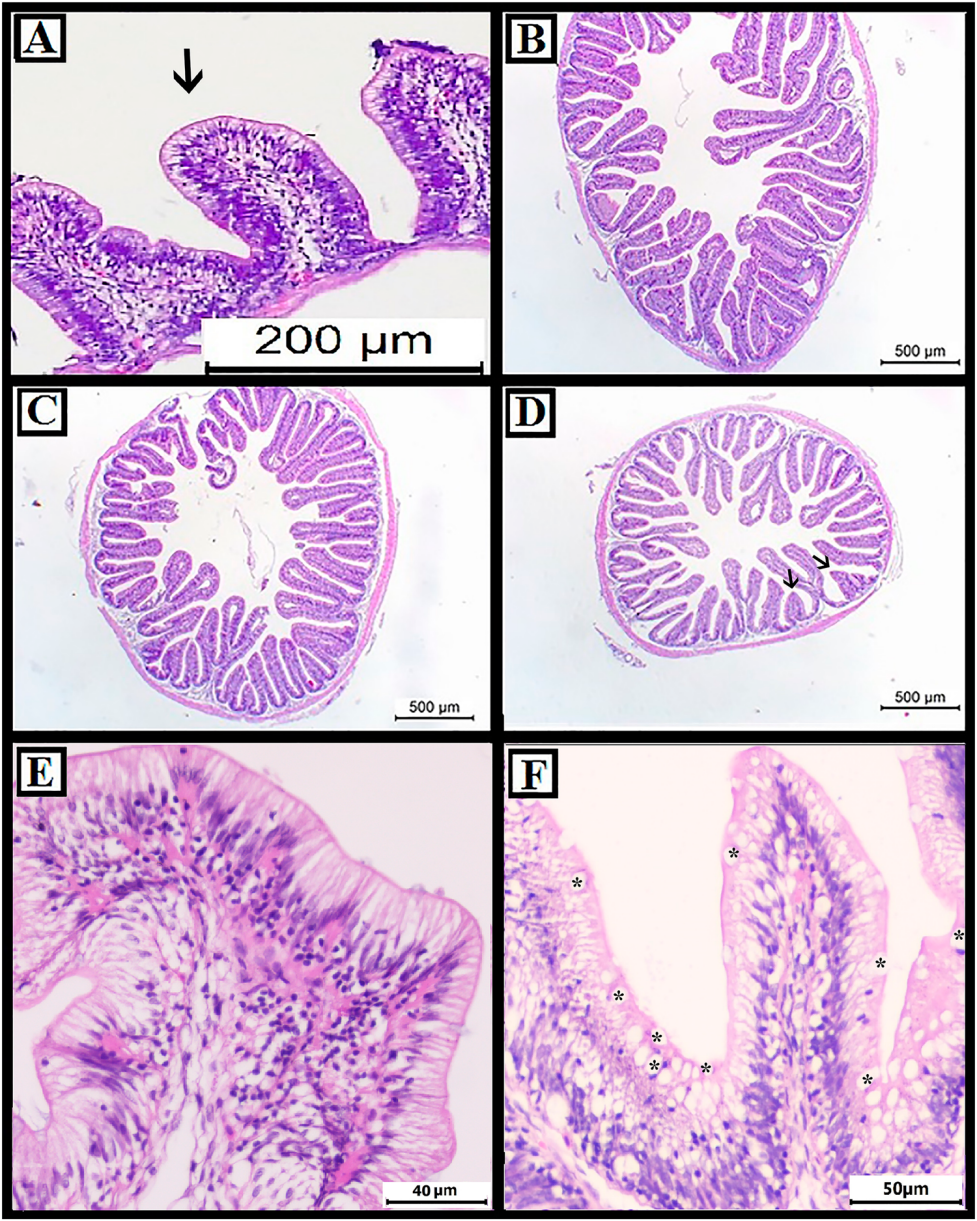
Histomorphology of the gut with different treatments after 15 days of experimental infection. Shrinkage of intestinal villi are shown using black arrow. Goblet cells are denoted by “*”. (**A**) G1 group (A-Live at 0.2%); (**B**) G2 group (A-Live at 0.2% and Aquaform at 0.2%); (**C**,**F**): G3 group (A-Live at 0.5% and Aquaform at 0.2%); (**D**,**E**) PC group.

After 15 days of treatment with the products, a higher quantity of digesta was observed in groups G2 and G3, suggesting that it might be owing to the higher feed intake observed in these groups (Supplementary Fig. 3). Autolysis was observed in the apical region of the intestinal villi in G2 and G3 groups, probably because of the presence of lumen ingestion since these groups had a higher feed consumption (Supplementary Fig. 3). In group G3, hyperplasia of goblet cells was observed (Fig. 3F). After infection, aggregates of lymphoid cells were observed in the PC group (Fig. 3E) In G1, shrinkage of some intestinal villi was visualized (Fig. 3A). No evident variations in the villi such as diffused shrinkage was found in groups G2 and G3 villi (Fig. 3B,C).

### Blood and innate immune parameters

The blood parameters are shown in Table 2. Some of the indicated characteristics are the significant increase (p < 0.05) in leukocytes and neutrophils after infection in the PC group compared to those in the G1 group. Before infection, there was a significant decrease (p < 0.05) in the number of erythrocytes (in G2 and G3), thrombocytes (in G1), hemoglobin (in G2), and monocytes (in G1, G2, and G3) compared to those in the NC group. Hematimetric indices and glucose showed no difference (p > 0.05) among the groups before or after infection.

**Table 2:** Blood general parameters (mean ± standard error) of experimental groups of fish supplemented with additives before and after disease challenge with *F*. *noatunensis* subsp. *orientalis* strain F1.

Analyzed period	Pre-challenge				Post-challenge			
Groups	NC	G1	G2	G3	PC	G1	G2	G3
Hematocrit (%)	39.6 ± 3.58	29.0 ± 0.79	31.2 ± 1.82	27.2 ± 2.36	31.4 ± 1.24	34.8 ± 0.14	31.2 ± 0.13	27.8 ± 0.18
Hemoglobin (g/dL)	6.34 ± 0.17^a^	5.73 ± 0.08^ab^	5.25 ± 0.13^b^	5.68 ± 0.08^ab^	4.98 ± 0.18	5.92 ± 0.59	4.66 ± 0.56	4.7 ± 0.77
Erythrocytes (10^6^/µL)	1.96 ± 0.07^a^	1.68 ± 0.02^ab^	1.23 ± 0.04^b^	1.35 ± 0.06^b^	1.86 ± 0.08	1.91 ± 0.07	1.43 ± 0.02	1.43 ± 0.04
Thrombocytes (10^3^/µL)	32.20 ± 1.13^ab^	27.90 ± 1.69^b^	Ag.	38.95 ± 0.63^a^	28.15 ± 1.46	30.25 ± 0.96	27.61 ± 1.33	27.86 ± 0.93
Leukocytes (10^3^/µL)	53.79 ± 2.04	55,12 ± 1.58	51.75 ± 0.91	39.34 ± 2.83	64.12 ± 0.32^a^	51.56 ± 1.21^b^	55.10 ± 1.87^ab^	53.16 ± 1.01^b^
Lymphocytes (10^3^/µL)	28.89 ± 1.03	30.54 ± 0.67	30.05 ± 0.82	24.22 ± 1.65	31.10 ± 0.38	30.50 ± 0.55	30.18 ± 1.21	29.32 ± 0.89
Neutrophils (10^3^/µL)	21.49 ± 0.98	23.30 ± 0.85	21.49 ± 0.74	14.62 ± 1.40	29.70 ± 0.97^a^	20.47 ± 1.20^b^	24.04 ± 1.15^ab^	22.63 ± 0.28^ab^
Monocytes (10^3^/µL)	3.04 ± 0.25^a^	0.84 ± 0.27^b^	0.19 ± 0.08^b^	0.35 ± 0.13^b^	0.81 ± 0.23	0.55 ± 0.16	0.72 ± 0.20	1.20 ± 0.21
Eosinophils (10^3^/µL)	0.36 ± 0.13	0.19 ± 0.09	0 ± 0	0,10 ± 0.03	0 ± 0	0 ± 0	0 ± 0	0 ± 0
Basophils (10^3^/µL)	0 ± 0	0.23 ± 0.12	0 ± 0	0.03 ± 0.01	0 ± 0	0.04 ± 0.02	0.12 ± 0.03	0 ± 0
MCV (fL)	203.39 ± 16.01	173.13 ± 4.23	272.13 ± 26.32	221,87 ± 27.42	172.48 ± 8.97	183.91 ± 5.67	222.61 ± 11.14	201.47 ± 10.43
MCHC (g/dL)	19.28 ± 2.50	19.91 ± 0.45	17.70 ± 1.10	23.63 ± 2.08	15.97 ± 0.28	15.64 ± 0.39	15.69 ± 0.80	16.86 ± 0.38
Glucose (mg/dL)	30.15 ± 1.28	31.57 ± 2.66	31.65 ± 2.76	43.29 ± 3.20	122.58 ± 13.26	91.50 ± 6.78	80.16 ± 7.25	76.31 ± 9.90

^*^Different letters (a and b) indicate significant difference between the treatments (p < 0.05); *Ag = Aggregated. NC, negative control; PC, positive control; G1, 0.2% A-Live treatment; G2, 0.2% A-Live and 0.2% Aquaform treatment; G3, 0.5% A-Live and 0.2% Aquaform.

Lysozyme activity was higher in G3 group before infection. However, after infection all groups that had contact with *Francisella* spp. showed an increase in this parameter but the difference among them was not significant (p > 0.05) (Table 3). Before infection, serum antibacterial activity was only present in G2 and G3, demonstrating that the combination of products stimulated this immunological parameter (Table 3).

**Table 3:** Data of serum lysozyme activity, complement activity and antibacterial activity (mean ± standard error) of fish from different groups of treatment before and after disease challenge with *F*. *noatunensis* subsp. *orientalis* strain F1.

Analyzed period	Pre-challenge				Post-challenge			
Groups	NC	G1	G2	G3	PC	G1	G2	G3
Lysozyme (µg/mL)	8.77 ± 0.25^b^	9.24 ± 0.25^ab^	9.43 ± 0.28^ab^	11.02 ± 0.19^a^	16.76 ± 0.42	17.18 ± 0.36	17.82 ± 0.43	16.73 ± 0.55
Complement activity mean (µL for lysis of 50% of erythrocyte)	2.11 ± 0.23^a^	0.82 ± 0.11^b^	0.98 ± 0.09^ab^	1.15 ± 0.08^ab^	1.07 ± 0.05	1.37 ± 0.09	1.09 ± 0.10	1.06 ± 0.13
Antibacterial activity of serum (Log2 mean of dilution + 1)	0 ± 0	0 ± 0	0.32 ± 0.15	0.63 ± 0.19	0 ± 0	1.27 ± 0.29	0.63 ± 0.19	0.95 ± 0.31

^*^Different letters (a and b) indicate significant difference between the treatments (p < 0.05).

NC, negative control; PC, positive control; G1, 0.2% A-Live treatment; G2, 0.2% A-Live and 0.2% Aquaform treatment; G3, 0.5% A-Live and 0.2% Aquaform.

### Microbiome diversity and abundance of the bacterial population

**Figure 4:**
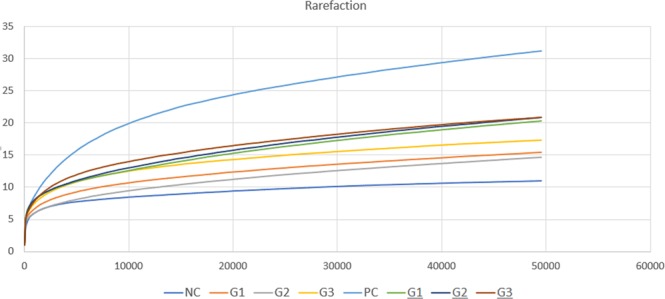
Rarefaction curve showing increasing species along the number of reads in different trial groups.

A total of 1,188,301 sequences were obtained for all groups, and 72 OTUs (operational taxonomy units) were identified. The number of sequences per group ranged from 88 to 182 thousand reads. A rarefaction curve showed that sequencing was sufficient to sequence most of the bacterial species present in the fish gut as there was little addition of new species after 10,000 sequences (Fig. 4), suggesting that the read count of the trial was representative of the intestinal bacteriome in all groups. Additionally, Supplementary Table 1 displays the total number of sequences obtained in each group.

**Figure 5:**
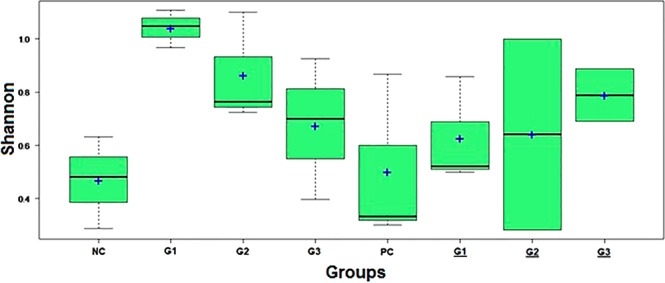
Shannon index in different trial groups after 15 days of treatment and after experimental infection with *Francisella* spp. (underlined).

Moreover, Mothur software was used to calculate the Shannon index, which compares the diversity of species in each group (Fig. 5). However, no significant differences were observed. The boxplot shows that NC and PC groups presented the lowest level of species diversity, while the treated groups had higher level of diversity. Thus, this information suggests that before and after infection by *Francisella* spp., the treated groups had higher diversity in the gut bacterial microbiome than that in the NC and PC groups. Groups G1 and G2 presented more bacterial diversity before infection in comparison to the bacterial diversity of groups G1 and G2 after infection by *Francisella* spp. Among all the groups, G1 was the most diverse; however, it showed a decreased diversity after 15 days of infection. Interestingly, after infection, the group (G3) that received a higher concentration of both products maintained a higher diversity at this time of the trial.

**Figure 6:**
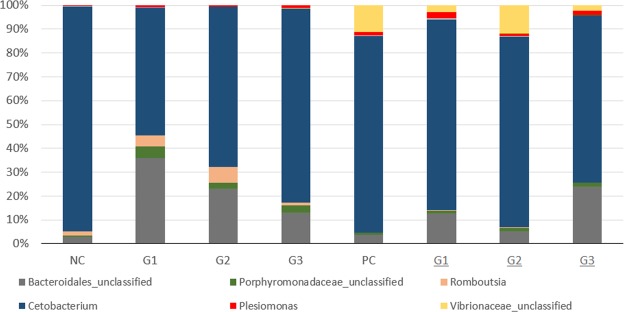
Abundance of noninfected and infected groups (underlined) with information on the percentage of sequences in each group. *Cetobacterium* (dark blue), Bacteroidales_unclassified (gray), Vibronaceae_unclassified (yellow), Porphyromonadaceae_unclassified (green), *Romboutsia* (pink) and *Plesiomonas* (red).

The abundance of bacterial species calculated by Mothur software is shown in Fig. 6 and Table 4. The abundance plot displays the most abundant bacteria in each sample: *Cetobacterium*, *Bacteroidales*, *Vibronaceae*, *Porphyromonadaceae*, *Romboutsia* and *Plesiomonas*. Before infection, in the NC group, the genus *Cetobacterium* was the most abundant, and the other families and genera (*Bacteroidales*, *Porphyromonadaceae*, *Romboutsia* and *Plesiomonas*) appeared in smaller numbers. On the other hand, in the treated groups, there was a decrease in the genus *Cetobacterium* in the gut microbiota. After infection, genus *Cetobacterium* remained in greater proportions in the groups, also favoring the increase in the genus of the family *Vibrionacea* (mainly in the PC and G2 groups).

**Table 4:** Count of sequences of the most abundant species in experimental groups.

Analyzed period	Pre-challenge numbers of sequences (%)				Post-challenge numbers of sequences (%)			
Taxon	NC	G1	G2	G3	PC	G1	G2	G3
*Cetobacterium*	46733 (94.2)	97834 (53.5)	112968 (67.3)	223726 (81.1)	105808 (82.5)	112245 (79.9)	99783 (79.9)	61951 (70)
Bacteroidales_unclassified	1486 (3.0)	65636 (36)	38781 (23.1)	36063 (13.1)	4969 (3.9)	17860 (12.7)	6612 (5.3)	21196 (24)
*Vibrionaceae*	0(0)	1(0)	1(0)	0(0)	14373 (11.2)	3879 (2.8)	14726 (11.8)	2077 (2.3)
Porphyromonadaceae_unclassified	207 (0.4)	8756 (4.8)	4218 (2.5)	8131 (3)	833 (0.6)	1712 (1.2)	1654 (1.3)	1445 (1.6)
Romboutsia	876 (1.8)	8419 (4.6)	10937 (6.5)	3477 (1.3)	22 (0.01)	239 (0.2)	460 (0.4)	55 (0.06)
*Plesiomonas*	187 (0.4)	1561 (0.8)	833 (0.5)	3221 (1.2)	1645 (1.3)	3732 (2.7)	1260 (1)	1499 (1.7)
Total of reads	49560	182643	167955	275745	128325	140541	124925	88543

NC, negative control; PC, positive control; G1, 0.2% A-Live treatment; G2, 0.2% A-Live and 0.2% Aquaform treatment; G3, 0.5% A-Live and 0.2% Aquaform.

**Figure 7:**
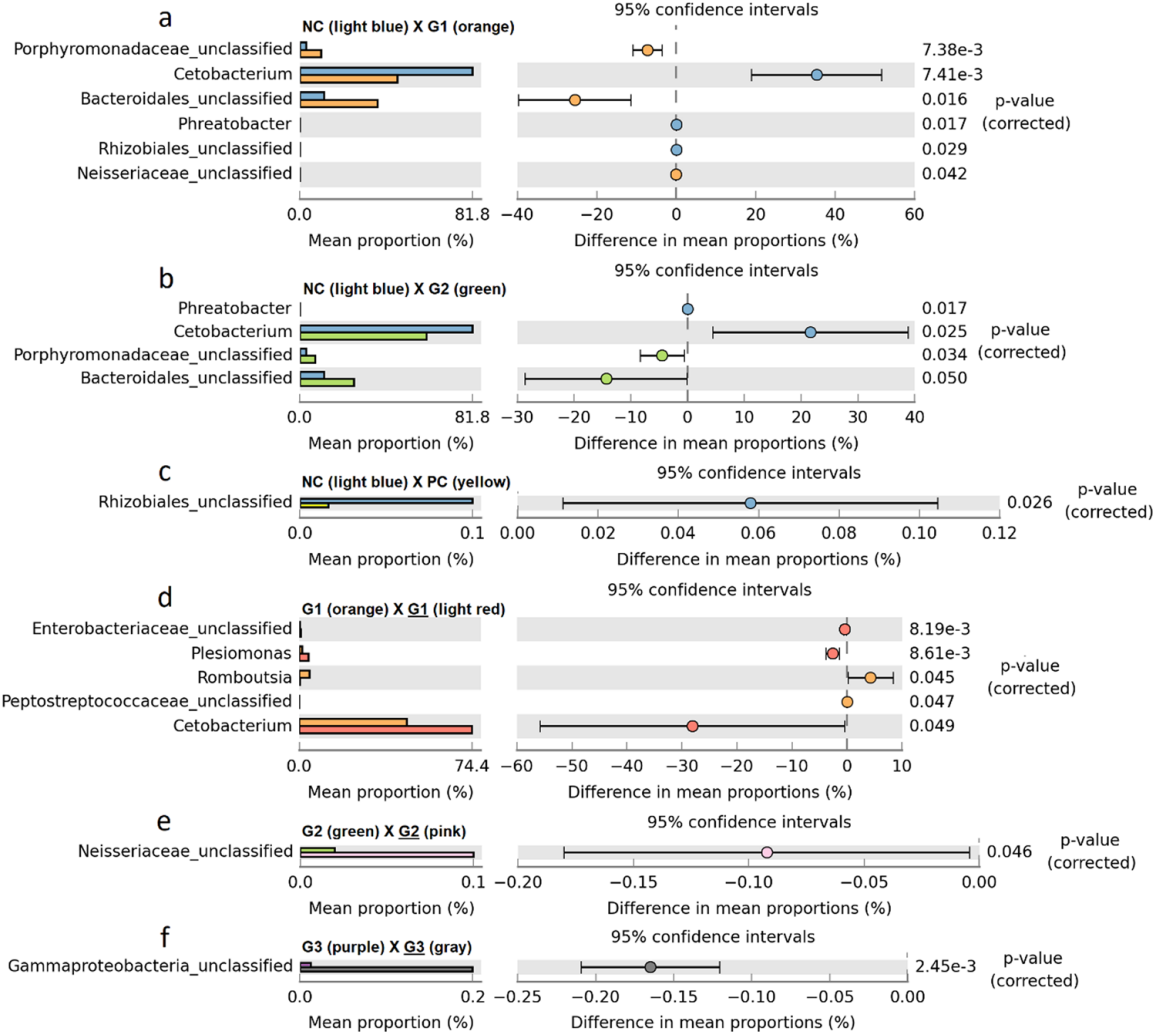
Comparisons of bacterial abundance among groups. The left side presents the abundance proportion of each sample. The right side shows the difference in abundance within 95% confidence intervals with the p-value of the significance test. Only significant results are displayed (p < 0.05).

STAMP analysis revealed significant difference in the proportion in the abundance results (Fig. 7). Thus, even small counts of sequence that are not seen in abundance plots are considered in STAMP analysis. Comparison of NC with G1 (Fig. 7a) showed significance in abundance of *Porphyromonadaceae*_unclassified, *Cetobacterium*, and *Bacteroidales*_unclassified. The *Porphyromonadaceae*_unclassified and *Bacteroidales*_unclassified considerably increased in the G1 group. On the other hand, *Cetobacterium* showed a decrease in abundance in the same group. *Phreatobacter*, *Rhizobiales*_unclassified and *Neisseriaceae*_unclassified were significant in the plot. Figure 7b displayed similar results, with discreet variations between the compared groups. The comparison of NC with G3 was not significant for any level of taxonomy. In Fig. 7c, the *Rhizobiales*_unclassified taxon displayed a large decrease in the PC when compared with the NC; however, there was a large standard deviation. The bacterial abundance in the infected groups (G1, G2 and G3) did not present any significant difference when compared to the bacterial abundance of the PC group. A comparison of the groups over time (before and after infection) showed different taxon among them (Fig. 7d–f). A higher number of *Cetobacterium* and *Plesiomonas* was observed in G1 (Fig. 7d), but the *Romboutsia* number decreased after infection (G1). Figure 7e displays an increase in *Neisseriaceae*_unclassified sequences in G2; however, this result presented a large standard deviation. Finally, Fig. 7f shows a large increase in the G3 group of *Gammaproteobacteria*_unclassified.

## Discussion

The products demonstrated very promising *in vitro* as well as *in vivo* results, with the combination of the products providing better benefits. *In vitro* data showed an inhibitory halo (for *Francisella* spp.) of 15 or 18 mm when using Aquaform and A-Live in combination, each at a low concentration (0.1%). Electron microscopy showed that the combination of 1% products resulted in a drastic reduction in the bacterial cell counts when compared with the other concentrations. Similar results were found in experiments using *Aeromonas* spp. and that there were inhibitory halos from a mixture of formic acid, propionic acid and calcium propionate^25^.

*In vivo* results showed better FCR and SGR in groups G2 and G3, which received the combination of the two products at A-Live concentrations of 0.2% and Aquaform of 0.2% and at A-Live of 0.5% and Aquaform of 0.2%, respectively. Mortality after infection was smaller in all groups receiving the products than in the positive control group. Additionally, mortality rate was significantly lower in the fish challenged with *Francisella* spp. by immersion than in the positive control group. These results reinforce that future studies with nutritional additives should use immersion challenge method, which resembles the natural infection, to obtain a more reliable data.

The autopsy analysis of the G2 and G3 groups showed that 15% and 35% of the animals, respectively, did not present characteristic lesions of francisellosis. These results along with immunity parameters and mortality data suggest that these high efficacy products can be an alternative strategy for prevention of francisellosis outbreaks in tilapia. In addition, the interference of products in pathogen homeostasis should also be explored in future studies through global gene expression and proteomic approaches. A previous study on tilapias showed an increase in weight gain, specific growth rate and apparent protein digestibility^31^. Additionally, the authors found a decrease in mortality rate (*Aeromonas hydrophila* challenge) and increase in serum lysozyme and phagocytic activity of the group treated with concentration of 0.3% potassium diformate. They also reported a decrease in intestinal pH in the treated groups, which resulted in increased beneficial bacteria count in the intestinal gut. Similarly, the innate immunity in current study, demonstrated a higher antibacterial and complement activity besides lower mortality in treated groups. Although intestinal pH is not assessed, the beneficial effects in fish in the present study may be associated with the antimicrobial effect of Aquaform (potassium diformate) in the intestinal tract by increasing intestinal health and nutrient absorption. In addition, it also suggested a synergistic effect with the A-live, which may have provided a positive effect to the intestinal and overall health of the animals even though no noticeable differences were observed in microbiome data. This hypothesis is supported in the present study in groups G2 and G3 data, both before and after the challenge, and in the *in vitro* results.

In the histological analysis of the intestine, it was observed that, post infection, the PC group presented aggregates of lymphoid cells and obtained low performance indices (growth and survival). On the other hand, the G2 and G3 groups that presented better performance indices and had higher villi based on intestinal histology. Also, in G3 group hyperplasia of goblet cells was observed. These results corroborate those found in other studies, where supplementation of organic acids in the diet improves mucus^32^ and nutrient digestibility in tilapia^33^.

Additionally, the use of products improved some blood and immunological parameters that may be related to the disease resistance in fish. The results of blood parameter analysis (mainly the increase in neutrophils in the PC group) indicated an acute inflammatory response to infection by *Francisella* spp., with the migration of neutrophils in large quantities to the site of infection resulting in the characteristic lesions of francisellosis in fish^34^. Alternately, in the treated groups, moderate increase in this type of immune cell may be due to an immunomodulation resulting from ingestion of the products, as shown in previous studies^25,35^. This immunomodulation hypothesis is reinforced by the results found in the G2 and G3 groups, with some fish presenting minor or even an absence of granulomatous lesions in the spleen. These two groups also showed the best results in FCR after infection, which may be related to reduction in the damage caused by the pathogen and an enhanced recovery of digestive functions. In addition, the product A-Live at the highest dietary concentration might have stimulated the innate immune system in different ways, which would explain the increase in the number of thrombocytes in the G3 group. The decrease in erythrocyte and hemoglobin values might be associated with physiological compensation, since they were within normal range for the species. Cytokine-related studies are needed to better understand the evolution of disease, pathogenicity and its relationship with the immune system in normal and treated fish. In addition, the active molecules harbored in A-Live (derivate from plant extracts) also should be explored. Research on the effect of purified bioactive molecules will aid in understanding the effect of these molecules, which may result in a more effective product for use in prevention of fish diseases. Glucose increased significantly in all groups after infection, which was expected since there is an increase in the hyperglycemic hormone cortisol during infections, which results in an increase in gluconeogenesis^36^.

Innate immunity parameters, in particular phagocytic, lysozyme and complement activities, have been used as indicators of the effects of inherent or external factors on the immune system and the disease resistance of fish. Among several factors that stand out are dietary and food additives^37,38^, such as immunostimulants and probiotics^39,40^, as well as the effects of diseases and vaccination^41,42^.

The level of serum lysozyme is an important index of innate immunity in fish. Lysozyme of fish having lytic activity against bacteria is well understood. Moreover, it has opsonic properties and helps to activate the complement system and phagocytes. Lysozyme activity is activated when the outer cell wall of Gram-negative bacteria bursts due to the action of complement and other enzymes that expose the inner peptidoglycan layer. The lysozyme then becomes effective and consequently increases its level and activity^43,44^. We observed in this study that, even before infection, the groups that were fed the products had higher lysozyme activity in comparison to the NC group, although this difference was not significant. When comparing the G3 group with the NC, we observed a significant increase in lysozyme activity. Post infection, all the groups had increased lysozyme activity. Thus, these results demonstrated that the products added to the diet displayed immunostimulatory role^25^, which suggest that dietary acidifiers have beneficial effect in aquaculture production assisting disease resistance.

Before infection, serum antibacterial activity was only present in G2 and G3 groups, indicating that the products stimulated this immune parameter. However, after infection all the treated groups (G1, G2 and G3) showed antibacterial activity. This result may be explained by the absorption and/or serum presence of active compounds in the diet or by the indirect stimulation of antibacterial serum components by the products in the feed which is quickly stimulated after a trigger (in this case, the infection by pathogen).

Before infection, we observed that all groups receiving the products showed better results of serum complement activity than that of the NC group. A significant difference in serum complement activity was observed in G1 group when compared to the serum complement activity of NC group. After infection, there was no significant difference between the groups, but G3 provided the best result. Similar results were found in other studies, where an improvement of the immune system from dietary supplementation has been reported^25,31^.

Studies have been conducted in vertebrates concerning host and intestinal microbiota interactions and demonstrated an integral role in ontogenetic development, especially when related to immune system modulation^45–47^. In addition to competition for readily available carbohydrates in the diet, intestinal microbes are able to extract energy from dietary polysaccharides that are indigestible by the host^48^.

In general, microbiome analysis showed a decrease in *Cetobacterium* and an increase in *Bacteriodales* in the treated groups before infection (Fig. 4). After infection, more discrete differences were observed in the microbiome between the groups (Table 4 and Supplementary Fig. 4). Further studies investigating the microbial composition of the fish gut are necessary to provide a comprehensive understanding of the influence of diet on the health status of fish. For example, the order *Bacteroidetes* was found in abundance in this study, as well as in marine herbivorous fish^49^ and other non-fish gut flora^50^.

The dominant phyla in the gut microbiome of Nile tilapia are generally *Proteobacteria* and *Fusobacteria*, although the relative abundances of these phyla vary between studies^51–54^. Microbiota analysis of tilapia gut in this study revealed *Cetobacterium* as the most prevalent genus in all intestinal samples. *Cetobacterium* has been recognized as a common member of the microbiome of grass, Asian bighead, common and Crucian carps^50,55–58^, and it may be considered a core genus among carps. This bacterium is a micro-aerotolerant anaerobe that is capable of producing vitamin B-12 and antimicrobial metabolites^59^. Thus, this interaction between pathogen and host suggests that tilapia may take advantage of the physiological benefits of this microorganism.

Our results are in agreement with a previous report^60^, which suggested that *C*. *somerae* is a commensal microbe with a highly abundant nucleus inside the intestine of catfish. Moreover, in the same study, the authors found the species *Plesiomonas shigelloides*, another bacterium belonging to the family *Enterobacteriacea* and commonly found in freshwater fish and aquatic environments. Additionally, *Plesiomonas shigelloides* is commonly found in the intestine of fish from tropical and subtropical countries such as Japan and Thailand, several countries in Africa, Australia and Brazil^61–64^. In this study, *Plesiomonas* spp. was present in all groups, even in small amounts. Considering that after infection only G1 group showed an increase in *Plesiomonas* spp. in the microbiota, and all remaining fish in this group presented spleen lesions from francisellosis, it was hypothesized that *P*. *shigeloides* played a role as a secondary/opportunistic pathogen in the disease evolution when group G1 showed worse result in feed conversion rate (FCR) than that of G2 and G3 groups.

Another genus that clearly appeared before infection was *Romboutsia*. This genus was previously known as *Clostridium*, and little information is available about this species^65^. Recently, a new species isolated from the small intestine of rats was described as *Romboutsia ilealis* CRIB^T^. Several studies have explored the link between the diet and gut microbiota due to the potential dietary properties presented by some species. The data shown here suggested that the products stimulated the increase in number of the genus *Romboutsia* before infection (groups G1 and G2 in Fig. 6). Additionally, an increase in *Bacteriodales* and an unclassified genus of the *Porphyromonadaceae* family was observed, suggesting a connection with a microbiota modulation observed in the treatment groups (as demonstrated in Fig. 7a,b).

The increase in the unclassified genus of the *Vibrionaceae* family in the infected groups might be related to the participation of this presumable pathogen in the symbiotic and commensal microbiota^60^. Interestingly, the group G3 after infection showed a small proportion of this bacteria family, suggesting an inhibitory effect of treatment against *Vibrionaceae* family. Fish with francisellosis are probably more susceptible to opportunistic pathogens, and an increase in this bacterium in the PC group suggests that francisellosis caused the fish to be more susceptible to secondary pathogens. It is noteworthy that minimal number of *Gammaproteobacteria* was detected after *Francisella* spp. infection, (Fig. 6). There are no reports about *Francisella* spp. pathogenesis affecting the fish gut; however, these results suggest that this pathogen may have few tropisms to this tissue.

There is global demand to reduce the use of antibiotics in animal production. Consequently, in the last decade, new alternatives such as acidifying products and plant extracts, have been widely explored for these economic activities, mainly in swine and poultry industries. More recently, the demand for these studies has increased in aquaculture, including fish farming. Accordingly, the data from this tilapia study indicated that the combination of Aquaform and A-Live presented the best results for growth, blood and immunological parameters and lower mortality in francisellosis-challenged model. Thus, the beneficial effects of these products on tilapia production were validated. Furthermore, studies on the effect of these additives on fish production are necessary to evaluate their efficacy against other diseases and/or stress conditions that may commonly occur in aquaculture.

## Materials and Methods

### Antibacterial activity of the products against *Francisella* spp

Addcon- Salus (Brazil) and MiXscience (France) provided the organic salt (Aquaform, potassium diformate) and vegetal-derived product (A-live, derived from plant extracts) used in this study, respectively. The tested phytogenic is a blend of plant extracts and mineral clays formulated to have high dispersion in water easily applicable in aquaculture. Antimicrobial activity of the tested phytogenic comes from sulfur organic compounds of the extracts from *Alliaceae* family (mainly garlic, onion, and leek in the present case). The *Alliaceae* family includes 13 genera and 600 species. Main representatives are onion, garlic, leek, shallots, and chives. The analysis consists of an *in vitro* bacterial growth inhibition test, which initially requires the bioactive compounds of the product to remain in aqueous solution. Thus, the solubility test of the products was performed using sterilized water, methanol (10, 20 and 30% in water) and ethanol (10, 20 and 30% in water). The best solvent (with low precipitation of both products) was 20% ethanol. The products were filtered using a 0.22 µm membrane. The products were tested at concentrations of 10, 5, 1, 0.5 and 0.1% individually and in combination; when combined, the ratio of the products was 1:1. *Francisella noatunensis* subsp. *orientalis* strain F1 was used at the 0.5 McFarland scale and plated on cystine heart agar supplemented with bovine hemoglobin (CHAH). Sterile discs were soaked separately with 10 µL of the different concentrations in duplicate. Petri dishes with a diameter of 9 cm were used, with two discs allocated for each dish. The plates were incubated at 28 °C for 72–96 h for further measurement of the inhibition halo, as previously described^25^.

### Electron microscopy of *Francisella* cells when exposed to products

This method aims to evaluate the possible physical damage caused by the products to the pathogen *Francisella* spp. using electron microscopy. After 72 h culture, colonies of the F1 strain were suspended in sterile saline, at the 1 McFarland scale, containing different concentration of products and incubated at 28 °C for 30 min. The samples were centrifuged for 5 min at 2000 rpm, resuspended in 100 μL of fixative (2.5% glutaraldehyde and 2% paraformaldehyde in 0.1 M sodium cacodylate buffer, pH 7.0) and transferred to 24-well polystyrene microtiter plates (Fisher Scientific, Kamstrupvej, Roskilde, Denmark) with glass coverslips precoated with a thin layer of poly-L-lysine (Sigma, Saint Louis, MO, USA). After 1 h, the volume was adjusted to 500 μL using fixing solution to avoid cell adherence to the coverslips and incubated at 25 °C for 12 h. The samples were post fixed in 1% OsO_4_ (Electron Microscopy Sciences, Washington, PA, USA) and dehydrated in an ethanol series (30, 50, 70, 90, and 100°GL). Samples were subjected to critical-point drying with CO_2_ BALTEC CPD 030 Critical Point Dryer (Shapiro Center for Engineering and Physical Science Research, New York, USA), coated with gold in BALTEC SDC 050 Sputter Coater (Capovani Brothers Inc., New York, USA) and observed under a scanning electron microscope (FEI Quanta 200, Netherlands).

### Fish

A total of 720 healthy juvenile Nile tilapia (*O*. *niloticus*) were obtained from a commercial hatchery from Paraná state, Brazil (mean individual initial weight was 34.3 ± 0.33 g). The fish were divided into 3 treatment groups (432 tilapia) distributed into 2 replicates (72 tilapia each) and 2 control groups (288 tilapia) distributed into 2 replicates (72 tilapia each). The animals were stocked in 150L tanks containing dechlorinated water with continuous renewal (80% of volume daily) and the temperature was maintained at approximately 25 °C, and the fish were fed three times a day until apparent satiation (resulting in approximately 6% ± 0.3% of biomass). Water parameters (pH of 6.8 to 7.2, total ammonia <0.4 mg/L, and absence of chlorine) were measured daily and maintained throughout the experimental period. The presence of oxygen was maintained by two aerators, resulting in dissolved oxygen of 5.4 mg/L on an average. Before the start of the feeding trial, the fish underwent a period of acclimatization for 7 days, and behavior/signs of disease were observed (exophthalmia, erratic swimming, skin lesions, and others). Microbiological diagnosis was also performed before the experiment, in which 20 fish were randomly sampled and euthanized by a high-dose of benzocaine (200 mg/mL). Aseptically, fragments of brain, liver, cranial kidney, and spleen were streaked on Mueller Hinton agar (Kasvi, São José dos Pinhais, PR, Brazil) enriched with 5% defibrinated ovine blood and in CHAH. The plates were incubated at 28 °C for 5 days for confirmation of the health status of fish (no bacterial growth in the plates). All animal procedures were approved by the Ethic Committee on Animal Use of State University of Londrina (Approval number CEUA/UEL-7327.2017.39) and all experiments were performed in accordance with relevant guidelines and regulations.

### Basal diet

An extruded feed (without organic acids and phytogenics) for early stage tilapia with 36% crude protein and 2.6 mm diameter was used. The nutrient composition of the diet is shown in Supplementary Table 2. For *in vivo* studies, the goal was to homogenously add the products to the feed, to enable it to reach and act locally at the digestive tract of the fish and for the absorption of bioactive molecules in the digestive tract. These steps were carefully carried out by sprinkling the products in a fractioned aqueous solution of the feed (one-third part of total volume for each time). The feeds were prepared separately using 5 kg of fed which was distributed in to a tiny layer on a plastic surface (5 meters on all four sides) on the floor. The first fraction of the products diluted in water was spread and the feed was mixed for 5 min. A similar process was followed to obtain the fraction two and three of the products.

In other words, phytogenic A-Live alone or in combination with Aquaform was incorporated in commercial feed by manual mixing for 5 min, followed by spraying the water-diluted products on the feed during agitation. Further, approximately 5% (w/v) of universal vehicle, a carboxymethylcellulose-based product (Vansil, Descalvado, SP, Brazil) was used for avoiding energetic/nutritional disbalance in the feed between the groups and to coat the surface of the feed and avoid rapid leaching of products in the water. In the feed of negative and positive control groups, only the carboxymethylcellulose product was added using the same steps. Then, the feed was spread on trays and dried for 12 h in an incubator at 35 °C before use.

### Experimental design and disease challenge

After acclimation, the fish were maintained under the same conditions until the end of experiment. Products were incorporated into the feed according to the experimental groups and provided daily for 15 days before the disease challenge. One day before the disease challenge, the water temperature was decreased gradually and maintained at 21 °C (±1 °C) to promote infection by *Francisella* spp., since outbreaks of francisellosis in Brazil occur at similar temperatures^14^. The experimental design is detailed below:

- NC- negative control group: No challenge with bacteria and no A-Live or Aquaform in the feed (2 tanks);
- PC- positive control group: Challenge with *Francisella* spp. and no A-Live or Aquaform in the feed (2 tanks);
- G1- group of fish that received A-Live at 0.2% in the feed for 15 days prior to experimental infection with the pathogen *Francisella* spp. (2 tanks);
- G2- group of fish that received the product A-Live at 0.2% and Aquaform at 0.2% in the feed for 15 days prior to experimental infection with the pathogen *Francisella* spp. (2 tanks);
- G3- group of fish that received the product A-Live at 0.5% and Aquaform at 0.2% in the feed for 15 days prior to experimental infection with the pathogen *Francisella* spp. (2 tanks).

The groups named G1, G2 and G3 received the products before experimental infection daily for 15 days and continued to receive the same dosage of the products after experimental infection (then named G1, G2 and G3) until the end of the experiment (at least 21 days after infection or when no mortality was observed for 3 days). After experimental infection, all the fish were observed daily for clinical signs and mortality. Dead fish were subjected to microbiological diagnosis by aseptically streaking cranial kidney and spleen fragments onto CHAH plates.

This experimental design was performed separately twice as different experiments and at different times. At first, the disease challenge was performed by feeding the fish (pathogen was administered at 8 × 10^6^ colony forming units (CFU)/g of feed for 24 h). In this first trial, the growth, blood parameters and mortality rate were evaluated. The second set of experiments were performed using an immersion challenge by diluting *Francisella* spp. in water (5.4 × 10^5^ CFU/mL of water in the tank). For *in vivo* trials using immersion challenge (that is closer to natural infection of fish), only the clinical signs, mortality, and presence of francisellosis lesions in kidney and spleen (and bacterial culture of these organs) were observed.

### Blood sampling

Blood samples were collected at 15 days posttreatment and 15 days post-challenge by *Francisella* spp. (five samples per group). The fish were anaesthetized with benzocaine (0.1 g/L), and blood was collected by puncturing the caudal vessel in 3 mL syringes (21 G) containing 10% anticoagulant (ethylenediaminetetraacetic acid). The blood was used to measure the hematocrit (Hct; %) using the microhematocrit method^66^, and red blood cells (RBC; 10^6^/µL) were counted in a Neubauer chamber following dilution at 1:200 in Dacie solution. White blood cell (WBC; 10^3^/µL) and total thrombocyte counts were calculated using an indirect method^67^. For differential counting of leukocytes, the smears were stained with May-Grünwald/Giemsa/Wright stain^66^. The hemoglobin concentration (Hgb; g/dL) was analyzed by the cyanmethemoglobin method^68^ using commercial kits (Labtest, Lagoa Santa, MG, Brazil) to determine the hematimetric indices of the mean corpuscular volume (MCV; fL) and mean corpuscular hemoglobin concentration (MCHC; g/dL). Glucose (Glu; g/dL) was measured using commercial kits (Labtest).

### Growth performance

Fish of all replicates were counted and weighed individually on the first and last day. The final body weight (FBW), weight gain (WG), daily weight gain (DWG) specific growth rate (SGR), and feed conversion ratio (FCR) were determined^69^.

### Innate immune analysis

The innate immune analysis was performed in all groups (five fish for each replicate) 15 days after treatment and 15 days after challenge with *Francisella noatunensis* subsp. *orientalis* strain F1. Blood samples were collected without anticoagulant, allowed to coagulate, and centrifuged at 1400 *g* for 10 min to obtain the serum, which was stored at −20 °C.

The serum lysozyme concentration was determined in triplicates using a method based on gram-positive *Micrococcus lysodeikticus* lysis^70^. Standard solutions (0–10 ng/μL) of chicken egg lysozyme L6876 (Sigma, Saint Louis, MO, USA) were prepared at the time of analysis from frozen aliquots for generating the standard curve. Subsequently, standard and 150 μL dilutions of the sample were placed in a small tube. A suspension of *Micrococcus lysodeikticus* M3770 (Sigma, Saint Louis, MO, USA) prepared in the same buffer was added to each tube. Absorbance were measured using a spectrophotometer 33D model (Coleman, Santo André, SP, Brazil) at 492 nm. The results were expressed using the values of the optical density variation for each sample volume versus the lysozyme volume of the standard curve. The linear regression equation of the standard lysozyme curve was used to determine serum lysozyme levels (ng/μL).

Alternative complement pathway activity (ACH50) was determined following the method using rabbit red blood cells (RaRBC) as target cells for hemolysis^71^. Briefly, serially diluted serums were mixed with rabbit erythrocyte (RaRBC) suspension and incubated at 25 °C for 1 h with occasional shaking. The extent of hemolysis was estimated by measuring the optical density of the supernatant at 490 nm (OD4,4). Serum dilutions resulting in greater than 90% or less than 15% lysis were excluded from the calculation and the serum dilution that resulted in 50% lysis of RaRBC was represented as ACH5O units/µL.

Antimicrobial activity was measured in a flat-bottom 96-well microplate. Fish serum was evaluated for its antimicrobial activity against *Escherichia coli* prepared at a concentration of 0.5 McFarland scale and diluted 100 times in Poor broth (PB). Subsequently, serial dilution of the serum in PB medium was performed at a factor of 1:2 for 12 dilutions. Saline solution diluted in PB was used as a control. Twenty microliters of the diluted bacteria were added to each well containing the serum and positive control. The microplates were incubated at 28 °C for 24 h. The serum antimicrobial activity was the reciprocal of the least dilution that showed bactericidal activity^72^.

### Intestine histology and morphology

Five animals from each experimental group (of the two replicates) were used in this analysis. Animals were euthanized by a benzocaine overdose of 250 mg/mL (previously diluted in 5 mL of ethanol and later diluted in water to obtain a final concentration of 250 mg/L in water). Digestive tract samples (distal to pylorus) from each experimental group were collected and then fixed in 10% buffered formalin solution and processed using routine methods. They were embedded in paraffin at 60 °C to obtain cross-sections with a thickness of 5 μm and stained with hematoxylin-eosin (H&E). The slides were mounted (Entellan, Darmstadt, Alemanha) and subjected to microscopic evaluation. The abundance of goblet cells within the villi and lymphoid cells were evaluated^73^.

### Statistical analysis

All statistical analyses were performed using R version 3.1.3 (R CORE TEAM, 2015). The growth performance, hematological analysis, glucose, and innate immune analysis were subjected to normality and homogeneity tests, followed by analysis of variance (ANOVA) and the Tukey’s test to compare arithmetic means, adopting a significance level of 5%. The data that did not present normality or homogeneity were transformed into log10 (x + 1), and the Tukey test was used at 5% significance. For quantitative variables that did not present a normal distribution even after transformation, the Kruskal-Wallis nonparametric test was used followed by the Dunn’s test with a significance level of 5%. The cumulative mortality was analyzed using the Fisher exact test with a significance level of 5% using OpenEpi v. 3.01 (https://www.openepi.com/Menu/OE_Menu.htm).

### Microbiome analysis

Nine fish from each group were used for the bacterial microbiome analysis. Each DNA sample was isolated from the stools of three fishes and pooled. After 15 days of treatment (to evaluate alterations in the bacterial microbiome due to product supplementation in feed) and 15 days after infection (to evaluate alterations in the bacterial microbiome after infection), the animals in each experimental group were euthanized by benzocaine overdosage. The stool of the entire intestinal tract was removed aseptically and maintained in sterile vials with refrigeration. The samples were immediately stored in a freezer at −80 °C until processing. For total DNA extraction, the commercial QIAamp DNA Stool Mini kit (QIAGEN, Hilden, Germany) was used according to the manufacturer’s instructions. Then, the V4 region of the 16S ribosome subunit gene^74^ was amplified with primers containing overlapping regions with Illumina platform primers. After verification of the amplicon quality, the samples were sent to Neoprospecta company for sequencing using the Illumina MiSeq (paired-end library) platform with the 250-cycle V2 kit. The bioinformatics analysis was performed using MOTHUR v.1.36.1 software^75^. Taxonomic classification was obtained using the SILVA database^76^. Briefly, the sequences were filtered by quality parameters (Phred value greater than 15, elimination of reads without their respective pair, among others) and removal of chimeras. The alpha diversity was calculated with MOTHUR software using the parameters Chao1 for richness and Shannon for diversity.

The statistical significance of alpha diversity parameters among groups was analyzed by ANOVA. Additionally, abundance plots were generated at the level of the genus, family and order using MOTHUR outputs. To verify the abundance significance of taxon between groups, STAMP (Statistical Analysis of Metagenomic Profiles) was performed using parent level 1 and profile level 6 to analyze the significance between two groups using the two-sided Welch’s t-test^77^.

Reads from sequencing were submitted to the FASTQ format for analysis using Mothur software v.1.39.5^78^ according to the MiSeq_SOP guidelines (https://www.mothur.org/wiki/MiSeq_SOP) to assemble 16S contigs and improve the quality of the sequences.

## Supplementary information

Supplementary figures and tables

## Data Availability

The authors declare that all information about this work are promptly available by contacting the corresponding author by email upaduapereira@uel.br. Also, all the raw data (file of reads in fastq format) are available in SRA database (www.ncbi.nlm.nih.gov/sra) with accession numbers SRR8585010 to SRR8585031).
