# Supplementary material for: Effects of a phytogenic, alone and associated with potassium diformate, on tilapia growth, immunity, gut microbiome and resistance against francisellosis
Source: Sci Rep. 2019 Apr 15;9:6045. doi: 10.1038/s41598-019-42480-8 (PMC6465292; doi:10.1038/s41598-019-42480-8)
Supplement: Supplementary file 1 — Supplementary figures and tables [file 41598_2019_42480_MOESM1_ESM.pdf]

Effects of a phytogenic, alone and associated with potassium diformate, on tilapia growth, immunity, gut microbiome and resistance against francisellosis

S. A. Suphoronski[1], R. T. Chideroli[1], C. T. Facimoto[1], R. M. Mainardi[1], F. P. Souza[2], N. M. Lopera-Barrero[2], G. F. A. Jesus[3], M. L. Martins[3], G. W. Di Santis[1], A. de Oliveira[4], G. S. Gonçalves[5], R. Dari[6], S. Frouel[6], U. P. Pereira[1*]

Supplementary Figure 1: Visualization of number of cells of *Francisella* sp. F1 strain after 30 minutes of exposure to different concentration of products separately, A (*Francisella* spp. without the products); B (*Francisella* spp. with Aquaform 0.5; C (*Francisella* spp. with Aquaform 1%), D (*Francisella* spp. with A-Live 0.5%); C (*Francisella* spp. with A-Live 1%).

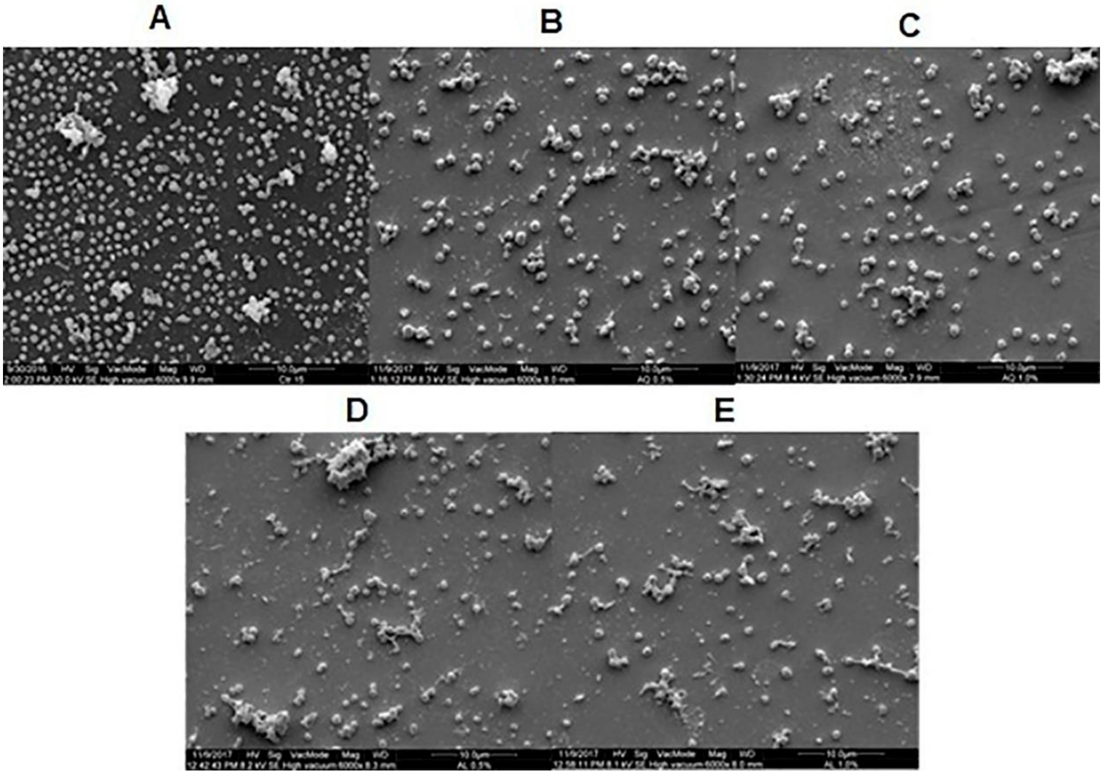

Supplementary Figure 2: Cumulative mortality observed in the different groups after disease challenge by the oral route with *Francisella noatunensis* subsp. *orientalis*.

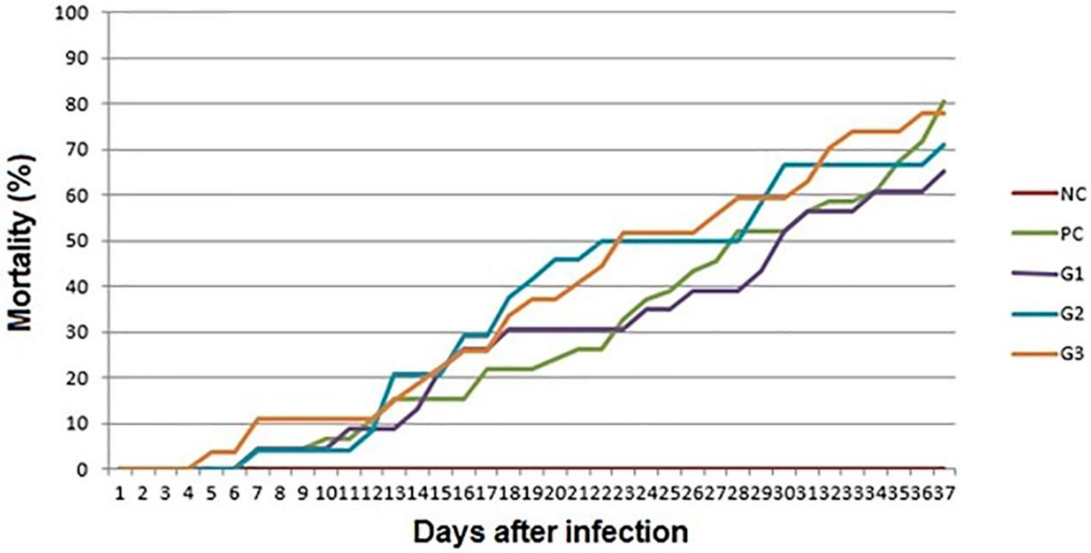

Supplementary Figure 3: Histomorphology of the gut with different treatments after 15 days of consumption of products in the feed. Presence of digest are shown using black arrow. Autolysis of apical region of villi are denoted by "*". (A) NC group; (B) G1 group (A-Live at 0.2%); (C) G2 group (A-Live at 0.2% and Aquaform at 0.2%); (D) G3 group (A-Live at 0.5% and Aquaform at 0.2%).

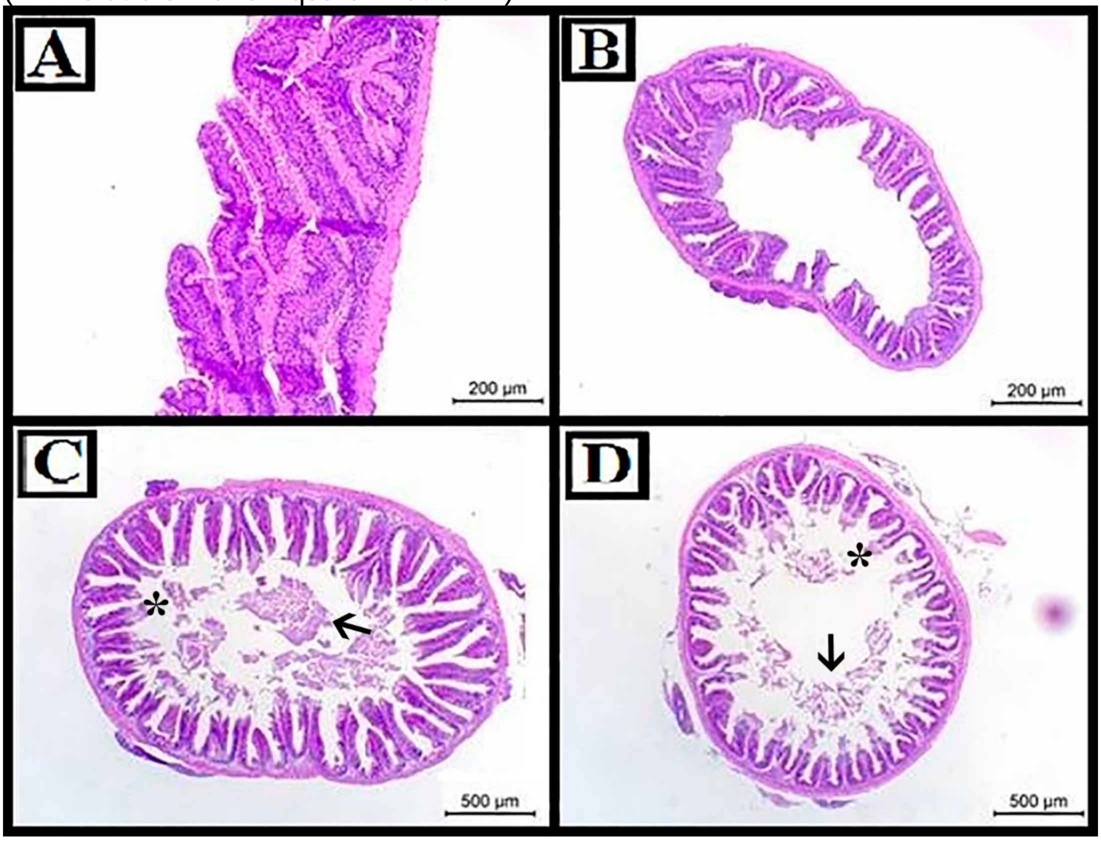

Supplementary Figure 4: Inverse Simpson index in different trial groups after 15 days of treatment (underlined) and after experimental infection with *Francisella* spp.

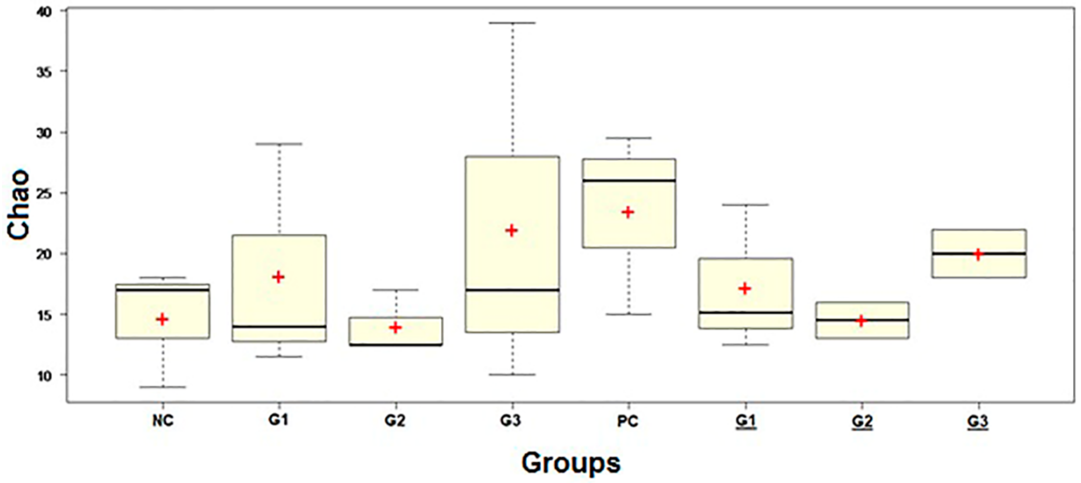

Supplementary Table 1: Count of sequences within group.

Analyzed period	Pre-challenge				Post-challenge			
Groups	NC	G1	G2	G3	PC	G1	G2	G3
Number of sequences	153597	182535	167909	181564	128368	160840	124945	88543

Supplementary Table 2: Nutrients composition (%) of the commercial fed used in the study.

Nutrients	Guarantee levels
Moisture (max)	120 g/kg
Crude protein (min)	360 g/kg
Ether extract (min)	70 g/kg
Fiber (max)	50 g/kg
Mineral matter (max)	140 g/kg
Phosphorus (min)	6000 mg/kg
Calcium (max)	25 g/kg
Calcium (min)	10 g/kg
Vitamin C (min)	350 mg/kg

Data provided by the manufacturer.
